# Roles of Vitellogenin and Its Receptor Genes in Female Reproduction of the Cigarette Beetle, *Lasioderma serricorne*

**DOI:** 10.3390/insects16020175

**Published:** 2025-02-06

**Authors:** Qian Guo, Mingxun Zu, Deqian Liu, Yi Yan, Wenjia Yang, Kangkang Xu

**Affiliations:** Key Laboratory of Surveillance and Management of Invasive Alien Species in Guizhou Education Department, College of Biological and Environmental Engineering, Guiyang University, Guiyang 550005, China; chinaguoqian7@126.com (Q.G.); 18212778602@163.com (M.Z.); ldqynzt@163.com (D.L.); yanheyi95@163.com (Y.Y.); yangwenjia10@126.com (W.Y.)

**Keywords:** vitellogenin receptor, vitellogenin, reproduction, cigarette beetle, RNAi

## Abstract

Vitellogenin (Vg) and the vitellogenin receptor (VgR) play essential roles in insect reproductive development. However, the functions of *Vg* and *VgR* genes in *Lasioderma serricorne*, an important insect pest of stored products, are unknown. We identified and characterized these two genes, designated *LsVg* and *LsVgR*, in *L. serricorne*. The open reading frames of *LsVg* and *LsVgR* were 5232 and 5529 bp, encoding 1743 and 1842 amino acid residues, respectively. Both *LsVg* and *LsVgR* were predominantly expressed in female adults and exhibited the highest expression in ovaries. The RNAi-mediated silencing of *LsVg* or *LsVgR* significantly decreased the average length of ovarian tubes and oocytes and severely affected ovarian development. The Knockdown of *LsVg* or *LsVgR* significantly reduced the oviposition period, the number of eggs laid, and the egg hatching rate. Females injected with ds*LsVg* and ds*LsVg + VgR* were found to had decreased vitellogenin content. The co-silencing of *LsVg* and *LsVgR* had a more pronounced effect on reducing the oviposition period and female fecundity in *L. serricorne*. This study revealed the importance of *LsVg* and *LsVgR* in regulating female reproduction and shows their potential as targets for RNAi-based control of *L. serricorne*.

## Simple Summary

Disrupting or blocking normal insect reproductive development is of great significance for developing new pest control strategies. In this study, vitellogenin (Vg) and its receptor (VgR) genes in *Lasioderma serricorne* were identified and functionally analyzed. *LsVg* and *LsVgR* are critical for regulating female reproduction. The RNAi-mediated knockdown of *LsVg* and *LsVgR* impaired ovarian development and significantly reduced fecundity and egg hatchability. These results suggest that targeting *LsVg* and *LsVgR* genes may help manage *L. serricorne* populations.

## 1. Introduction

The cigarette beetle, *Lasioderma serricorne* (Fabricius), is an important storage insect pest found worldwide. *L. serricorne* damages stored products, including tobacco, cereals, traditional Chinese medicines, and many plant and animal materials [1]. *L. serricorne* has a high reproductive ability and can rapidly develop into a large population. The ovarian development of *L. serricorne* begins in the pupal stage, as adults can mate 24 h after emergence. Females can begin to lay eggs within 12 h and lay more than 100 eggs at their peak [1,2]. However, there are few studies on the genetic basis of the reproductive development of *L. serricorne*. The RNA interference (RNAi)-mediated silencing of an insulin signaling pathway gene *Akt* in *L. serricorne* in a previous study led to the obstruction of the ovaries and decreased female fertility [2]. Two microRNA pathway genes (*LsDicer-1* and *LsArgonaute-1*) regulate the development of follicular epithelial cells by influencing key miRNAs, thus, delaying the oocyte development of *L. serricorne* [3]. These findings suggest the interference of reproduction as a pest management strategy.

Female reproduction is a crucial factor for the maintenance and continuation of insect populations [4]. The inhibition of female fertility can significantly restrict population growth and reduce the economic losses caused by these pests [5]. Therefore, disrupting or blocking the normal reproductive development of insects using RNAi is an active research field. The delivery of dsRNA to insects is a crucial component of this strategy. One efficient delivery method is through transgenic plants engineered to express pest-specific dsRNA [6]. When pests feed on these plants, they ingest the dsRNA, which subsequently activates the RNAi pathway. A previous study demonstrated that transgenic corn expressing the dsRNA of the *Hunchback* gene effectively suppressed field populations of *Diabrotica virgifera virgifera*. This suppression was attributed to reproductive failure and reduced offspring viability resulting from the RNAi-mediated gene silencing [7]. Although challenges such as non-target effects and the potential development of resistance remain, using RNAi technology to control insect pests by manipulating their reproductive capacity provides an alternative to traditional pest control methods [8].

The reproductive system of arthropods offers a variety of control targets, with many important and unique genes represented. Vitellogenin (Vg) and the vitellogenin receptor (VgR) are involved in the reproductive processes of oviparous species, and Vg is the precursor of the major vitellin (Vn) in insects, while VgR mediates the endocytosis of Vg into oocytes [9,10]. Vg and VgR are crucial nutrients for developing embryos. The Vg and VgR-based reproductive systems of insects have been identified as targets for endocrine disruptors. For example, decreased expression levels of *SfVgR* or *SfVg* in *Spodoptera frugiperda* were found to obstruct oocyte maturation and significantly reduce fertility [11]. The functions of *Vg* and *VgR* genes in reproductive development have been verified in many insect families, including Lepidoptera [12], Coleoptera [13], Diptera [14], Orthoptera [15], Neuroptera [16], Hymenoptera [17], Blattaria [18], and Hemiptera [19]. In addition to its critical roles in insect reproduction, VgR can participate in the regulation of host searching [20] and virus transmission [21]; Vg also plays essential roles in host-seeking behavior [22], plant defense [23], antioxidation [24], immune response [25], and lifespan regulation [26].

In this study, we identified and cloned the full-length open reading frame (ORF) sequences of vitellogenin and its receptor genes (*LsVg* and *LsVgR*) in *L. serricorne*. We conducted phylogenetic analyses and multiple sequence alignments to compare LsVg and LsVgR proteins with those of other insect species. We analyzed the expression profiles of *LsVg* and *LsVgR* at different developmental stages and in various adult tissues. Finally, we used RNAi-mediated gene silencing to investigate the functions of *LsVg* and *LsVgR* in ovarian development and female fecundity of *L. serricorne*. These results will clarify molecular mechanism of *Vg* and *VgR* genes in the regulation of insect reproduction and provide new effective targets for controlling pests.

## 2. Materials and Methods

### 2.1. Insects

The *L. serricorne* used in this study were collected in 2014 from a tobacco warehouse (26°34′ N, 106°41′ E) in Guiyang City, Guizhou Province, China. The insects were reared on *Angelica sinensis* in incubators at 40% ± 5% relative humidity and 28 °C ± 1 °C in darkness (LD 0:24), as previously described [27].

### 2.2. Molecular Cloning and Sequencing of LsVg and LsVgR

Total RNA was isolated from *L. serricorne* adults using a TransZol reagent (TransGen Biotech, Beijing, China), and the integrity was further verified using agarose gel electrophoresis. The cDNA sequences of the *Vg* and *VgR* genes were obtained from the *L. serricorne* transcriptomic database (SRR13065789) and amplified with PCR using 2 × EasyTaq^®^ PCR SuperMix (+dye) Kit (TransGen Biotech) with gene-specific primers (Table S1). The PCR parameters were as follows: 94 °C for 3 min, 38 cycles of 94 °C for 30 s, 58 °C for 30 s, 72 °C for 4 min, and 72 °C for 10 min. The products were ligated into the pGEM-T Easy Vector (Promega, Madison, WI, USA) and were then sequenced using Tsingke Biotech (Chongqing, China).

### 2.3. Sequence and Phylogenetic Analysis

The molecular weight (MW) and isoelectric points (pI) were computed using ExPASy tools (http://www.expasy.ch/) (accessed on 5 January 2025). The signal peptides and structural domains of these homologous proteins were predicted using the SMART (http://smart.embl-heidelberg.de/) (accessed on 5 January 2025). The amino acid sequences were aligned using ClustalW (https://www.genome.jp/tools-bin/clustalw) (accessed on 5 January 2025). A phylogenetic tree was constructed using the neighbor-joining method in MEGA 7 software with 1000 bootstrap replications [28].

### 2.4. Spatio-Temporal Expression Analysis

The whole bodies of female beetles from 1-day-old pupae to 5-day-old adults (FP1–FP5 and FA1–FA5) were collected for developmental expression analysis. Each sample included at least 30 individuals, and three biological replicates were prepared per sample. Eight tissues from female adults, including the head, thorax, cuticle, leg, midgut, elytra, ovary, and hind wing, were dissected for tissue-specific expression analysis. Three biological replicates were set, and more than 50 individuals were pooled in each replicate. The qPCR was amplified by the TransStart^®^ Top Green qPCR SuperMix (TransGen Biotech). The amplification program comprised the following: 94 °C for 30 s, followed by 45 cycles of 94 °C for 5 s, 60 °C for 30 s, and 72 °C for 10 s. The amplification specificity was verified using melting curve analysis. All experiments were performed in triplicate, and the relative expression levels were calculated using the 2^−∆∆CT^ method [29]. The *elongation factor 1-alpha* (*EF1a*) and *18S ribosomal RNA* (*18S*) of *L. serricorne* were chosen as internal reference genes [30].

### 2.5. RNA Interference Bioassay

RNAi was used to explore the functions of *LsVg* and *LsVgR* in female reproduction. The dsRNA-specific primers were designed in dsRNAEngineer (https://dsrna-engineer.cn/) and listed in Table S1. The dsRNA was synthesized using a TranscriptAid T7 High Yield Transcription Kit (Thermo Scientific, Wilmington, DE, United States), before being purified using phenol/chloroform solution, precipitated using ethanol, and dissolved in nuclease-free water. Each 3-day-old female pupa was injected with approximately 200 ng of ds*LsVg*, ds*LsVgR*, or ds*LsVg* + *LsVgR* (mixture at a 1:1 ratio) using a Nanoliter 2010 microinjector (WPI, Inc., Sarasota, FL, USA). The dsRNA of *green fluorescent protein* (*GFP*)-injected pupae served as the negative control. At 3 and 5 d after dsRNA injection, 30 individuals were collected to detect RNAi efficiency using qPCR, and three biological replicates were prepared. The 5-day-old female adults, following dsRNA injection, were dissected to observe ovarian development using a stereomicroscope VHX-2000C, and the lengths of the ovarian tube and primary oocyte were measured. Newly emerged female adults, resulting from dsRNA-injected pupae, were collected within 12 h of eclosion for the mating assay. In a Petri dish, each female was paired with an untreated male (*n* = 30 for each of the three replicates) of the same age. The oviposition period and total number of eggs laid were recorded for each female until death by using a stereomicroscope, and hatching rates were counted. The Insect Vitellogenin Enzyme-linked Immunosorbent Assay (ELISA) Kit (Meilian Biotechnology Co., Ltd., Shanghai, China) was used to measure the vitellogenin content 5 days after dsRNA injection.

### 2.6. Statistical Analysis

All data were analyzed using SPSS 20.0 software (IBM Corp, Chicago, IL, USA) and were represented as the mean ± standard error (SE). Differences between the two treatments were compared using Student’s *t*-test at significance levels of ** *p* < 0.01 and *** *p* < 0.001. A one-way analysis of variance (ANOVA) followed by a least significant difference test was applied to compare differences among multiple samples.

## 3. Results

### 3.1. Sequence and Phylogenetic Analysis of LsVg and LsVgR

**Figure 1:**
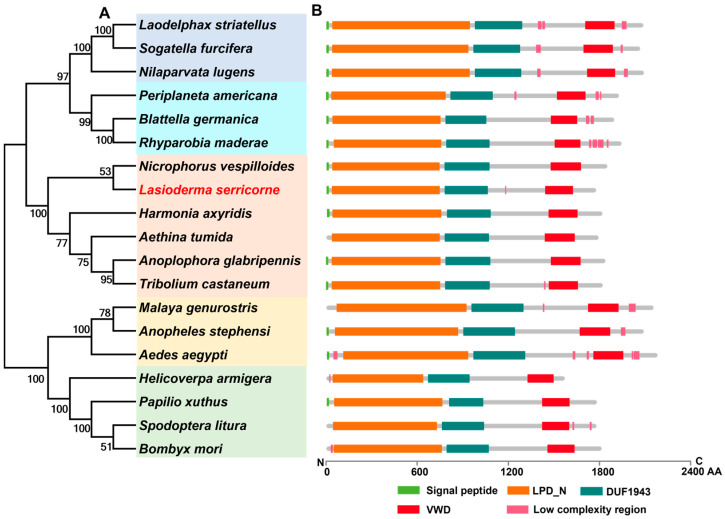
Phylogenetic tree (**A**) and structure comparison (**B**) of insect vitellogenin proteins. The GenBank accession number of each species is listed in Table S1. AA, amino acid; LPD_N, lipoprotein vitellogenin_N domain; DUF1943, domain of unknown function 1943; VWD, von Willebrand factor type D domain.

The ORF sequence of *LsVg* (GenBank accession number: UVT84831.1) consisted of 5232 bp, encoding 1743 amino acid residues, with an MW of 198.8 kDa and pI of 6.46. A signal peptide of 16 amino acids was found in the N-terminal end of the putative protein of *LsVg*. Multiple sequence alignment revealed that LsVg shared high conservation levels with other insects in the conserved regions of Vg proteins, including the lipoprotein vitellogenin_N domain (LPD_N, residues 18–729), domain of unknown function 1943 (DUF1943, residues 761–1044), von Willebrand factor type D domain (VWD, residues 1422–1605), and low-complexity region (Figure 1). The homology analysis showed that LsVg shared 39.4% and 35.9% of its identity with the Vg of *Nicrophorus vespilloides* (XP_017781017.1) and *Anoplophora glabripennis* (XP_018565741.1), respectively. The phylogenetic analysis of Vg from different insect species showed that LsVg clustered with the branch of Coleoptera species and was most closely related to *N. vespilloides* (Figure 1).

**Figure 2:**
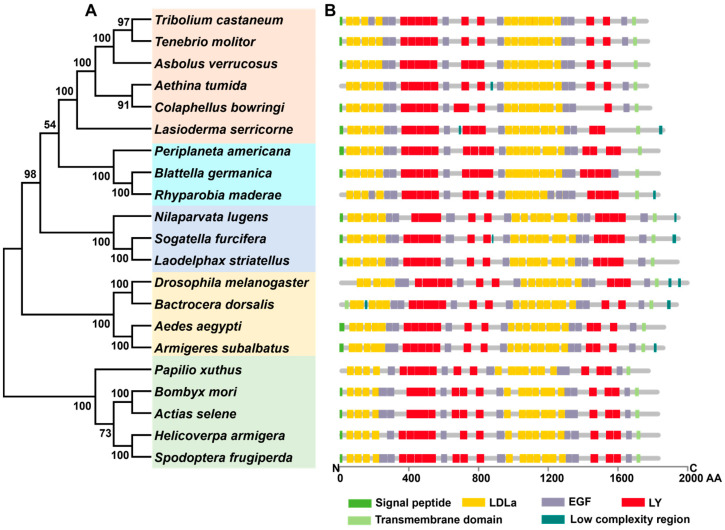
Phylogenetic tree (**A**) and structure comparison (**B**) of insect vitellogenin receptor proteins. The GenBank accession number of each species is listed in Table S1. AA, amino acids; SP, signal peptide; EGF, epidermal growth factor-like domain; LdLa, low-density lipoprotein receptor domain class A; LY, low-density lipoprotein receptor YWTD domain.

The ORF sequence of *LsVgR* (GenBank accession number: UVT84832.1) consisted of 5529 bp, encoding 1842 amino acid residues, with a MW of 207.1 kDa and pI of 5.11. The predicted signal peptide of LsVgR was 21 amino acids at the N-terminus. Multiple sequence alignment showed that LsVgR was a typical transmembrane protein with several conserved domains, including the low-density lipoprotein receptor domain class A (LDLa), epidermal growth factor-like domain (EGF), low-complexity region, and low-density lipoprotein receptor YWTD domain (LY). LsVgR had five class A LDLa repeats at the N-terminal and eight repeats in the middle of the putative protein sequence. The homology analysis showed that LsVgR shared 46.6% and 45.1% of its identity with the VgR of *A. glabripennis* (XP_018579962.1) and *N. vespilloides* (XP_017771582.1), respectively. Phylogenetic analysis showed that LsVgR clustered with the VgR of other Coleoptera species, indicating high sequence similarity (Figure 2).

### 3.2. Spatio-Temporal Expression of LsVg and LsVgR

**Figure 3:**
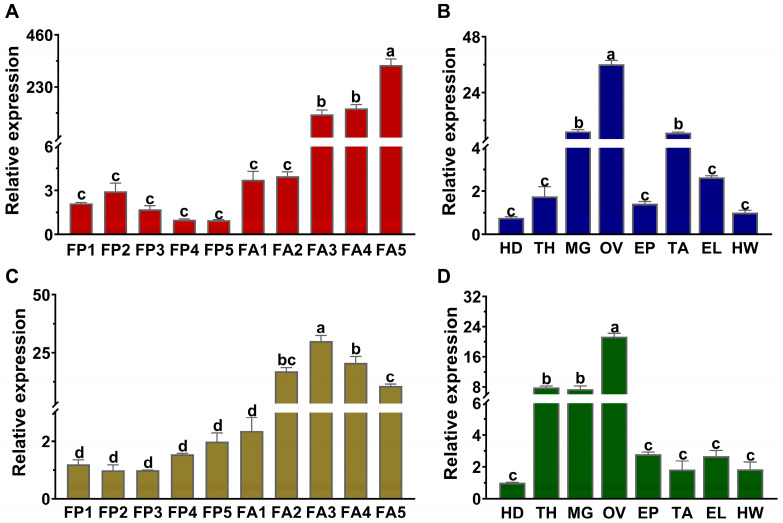
Spatio-temporal expression of *LsVg* and *LsVgR* in *Lasioderma serricorne*. Relative expression of *LsVg* in different developmental stages (**A**) and adult tissues (**B**). Relative expression of *LsVgR* in different developmental stages (**C**) and adult tissues (**D**). FP1–FP5, days 1–5 of female pupae; FA1–FA5, days 1–5 of female adults. TH, thorax; HD, head; OV, ovary; MG, midgut; TA, tarsus; EP, epidermis; EL, elytra; HW, hind wing. Different lower letters above bars indicate a significant difference derived from one-way ANOVA followed by the least significant difference test.

The results demonstrated that the expression profiles of *LsVg* and *LsVgR* were significantly different among various developmental stages and adult tissues (Figure 3). The highest expression level of *LsVg* was detected in 5-day-old female adults and lowest during the female pupal stages. Expression was 337-fold higher in 5-day-old female adults than in 5-day-old female pupae (Figure 3A). A similar expression pattern was seen for *LsVgR,* where the expression level in 3–5-day-old female adults was significantly greater than in pupae, and the expression of *LsVgR* in 3-day-old female adults was 30-fold higher than in 3-day-old female pupae (Figure 3C). Both *LsVg* and *LsVgR* were continuously expressed in all tested tissues but were most highly expressed in the ovary of female adults. In addition, *LsVg* was also mainly expressed in the midgut and leg, while *LsVgR* was highly expressed in the midgut and thorax (Figure 3).

### 3.3. Effects of RNAi on Ovarian Development of L. serricorne

**Figure 4:**
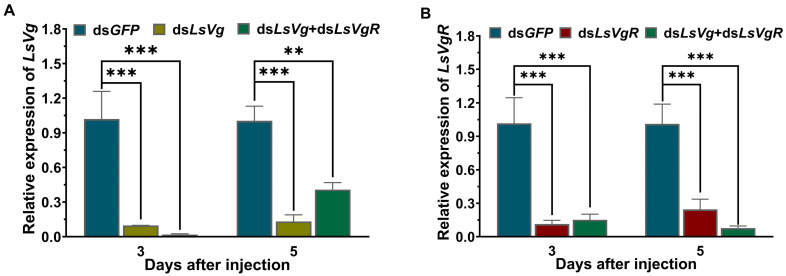
Relative expression levels of *LsVg* (**A**) and *LsVgR* (**B**) at 3 and 5 days after injection with ds*LsVg*, ds*LsVgR*, and ds*LsVg* + *VgR*. ds*GFP* was used as a control. Significant differences between the RNAi group and control groups were determined using Student’s *t*-test (** *p* < 0.01, *** *p* < 0.001).

**Figure 5:**
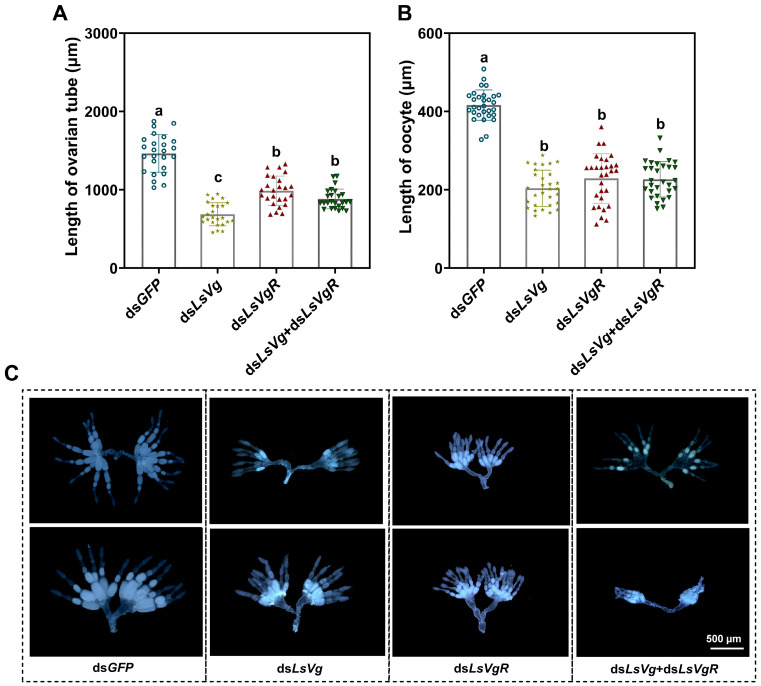
Effects of *LsVg*, *LsVgR*, and *LsVg* + *VgR* RNAi on ovarian development in *Lasioderma serricorne*. The lengths of the ovarian tube (**A**) and oocyte (**B**) after injection with ds*LsVg*, ds*LsVgR*, and ds*LsVg* + *VgR*; ds*GFP* was used as a control. Different lower letters above bars indicate a significant difference derived from one-way ANOVA followed by the least significant difference test. (**C**) Ovarian morphological changes. Ovaries were dissected and photographed at 5 days after adult eclosion.

The expression levels of *LsVg* and *LsVgR* were significantly decreased by 90.5% and 86.9%, and by 88.9% and 75.8% at 3 and 5 days after injection with ds*LsVg* and ds*LsVgR*, respectively, compared with the control group (Figure 4). In addition, the expression levels of *LsVg* and *LsVgR* were significantly downregulated after injection with the mixture of ds*LsVg* and ds*LsVgR* (ds*LsVg* + *VgR*). The average lengths of the ovarian tube in the ds*LsVg*, ds*LsVgR*, and ds*LsVg* + *VgR* injection groups were 687.0, 987.1, and 882.2 μm, respectively, which represented decreases of 53.0%, 32.5%, and 39.7% compared with 1463.4 μm in the dsG*FP*-injected control group (Figure 5A). The average oocyte lengths in the ds*LsVg*-, ds*LsVgR*-, and ds*LsVg* + *VgR*-treated groups were 203.7, 228.9, and 226.5 μm, respectively, which were significantly lower than the average length (416.5 μm) in the control group (Figure 5B). After injection with ds*LsVg*, ds*LsVgR*, and ds*LsVg* + *VgR*, the number and size of mature eggs were smaller than those of the control group, and most of the eggs had lower yolk deposition and were empty. The effect of co-silencing *LsVg* and *LsVgR* was the most significant (Figure 5C).

### 3.4. Effects of RNAi on Female Fertility of L. serricorne

**Figure 6:**
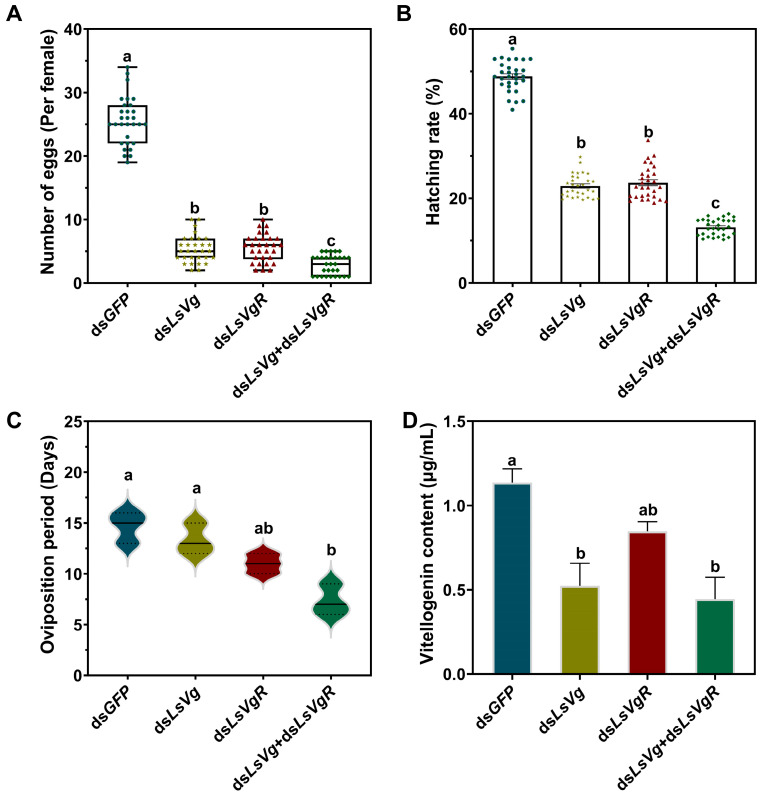
Effects of *LsVg*, *LsVgR*, and *LsVg* + *VgR* RNAi on female fecundity in *Lasioderma serricorne*. (**A**) The mean number of eggs laid per female after injection with ds*LsVg*, ds*LsVgR*, and ds*LsVg* + *VgR*; ds*GFP* was used as a control. (**B**) The hatching rate of offspring eggs. (**C**) Changes in oviposition period. (**D**) Determination of vitellogenin content. Different lower letters above bars indicate a significant difference derived from one-way ANOVA followed by the least significant difference test.

The silencing of *LsVg* and *LsVgR* caused an obvious decrease in female fecundity after injections of ds*LsVg*, ds*LsVgR,* and ds*LsVg* + *VgR*. The mean numbers of eggs laid per female in the ds*LsVg*-, ds*LsVgR*-, and ds*LsVg* + *VgR*-injected groups were 5.3, 5.5, and 2.9, respectively, which were significantly fewer than the mean of 25.5 eggs per female in the ds*GFP*-injected control (Figure 6A). The egg hatching rates in the ds*LsVg*, ds*LsVgR*, and ds*LsVg* + *VgR*-injected groups were significantly reduced by 53.1%, 51.4%, and 73.0%, respectively, compared with ds*GFP*-injected controls (Figure 6B). The oviposition period of females in the ds*LsVg* + *VgR*-injected group was shortened by 7.4 days compared with the control (Figure 6C). The vitellogenin content after ds*LsVg* and ds*LsVg* + *VgR* injection was significantly lower than that in the control group (Figure 6D).

## 4. Discussion

In this study, we obtained the full-length ORF sequences of *LsVg* and *LsVgR* genes in *L. serricorne*. The putative protein of *LsVg* contains DUF1943, LPD_N, and VWD domains, which are large and highly conserved domains in most insect species. The LPD_N domain is located at the N-terminus of LsVg and is associated with lipid transfer activity [31]. DUF1943 is considered to be immune-related: similar functions have been observed in the Vg protein of *Anopheles gambiae*, which plays a role in the anti-plasmodium response [32]. The amino acid sequence of *LsVgR* contains multiple LDLa, EGF, and LY domains, suggesting that this gene belongs to the LDLR gene family, which is involved in the receptor-mediated endocytosis of lipoproteins like Vg [10].

Since the expression of *Vg* and *VgR* is usually absent or relatively low in male insects [31,33], we focused on the expression of *LsVg* and *LsVgR* in *L. serricorne* females. The expression of these two genes was detectable in various tissues of female adults, and the highest expression level was found in the ovaries during the oviposition period. This is the most representative characteristic of *Vg* and *VgR*, which is related to their primary function in egg production [19]. The individual expression patterns of *LsVg* and *LsVgR* were highly consistent during the developmental and reproductive periods of *L. serricorne*. The co-expression model of *Vg* and *VgR* is commonly identified in other insect species. In *Thitarodes pui*, *VgR* and *Vg* are highly expressed in adult and pupal stages, and they are both enriched in the abdomen [34]. The transcript levels of *SfVgR* and *SfVg* in *Sogatella furcifera* were similar in females at different times after emergence. Vg was specifically enriched in the fat body, while VgR was enriched in the ovary [19]. These results showed that either *Vg* or *VgR* is indispensable for important physiological processes in insects. Besides the ovary, Vg was also found to be highly expressed in other tissues, such as the wing, gut, fat body, and central nervous system [12,35,36]. The expression of *Vg* in different tissues shows the diverse roles of this gene in various physiological processes of insects [37]. The existence of Vg in the honeybee brain indicated its involvement in the energy metabolism of glial cells [38]. In *Temnothorax longispinosus*, workers specializing in brood care exhibited a high expression of *Vg*, and young workers reduced their brood care activity upon the downregulation of *Vg* [39]. The expression of *LsVg* and *LsVgR* in other segments, such as the midgut and thorax, was also detected. This suggests they might play a role in midgut immune defense and nutrient transport.

RNAi was applied to explore the roles of *Vg* and *VgR* in the female reproductive system and to predict their potential as RNAi-based control targets [8]. A high silencing efficiency for dsRNA was required in RNAi assays. The qPCR analysis showed that, after injection, both ds*LsVg* and ds*LsVgR* had excellent silencing efficiency on the target genes, as did the mixture of both. Combined with a previous RNAi analysis of *L. serricorne*, these results indicate that injection is an effective laboratory method for dsRNA delivery to *L. serricorne* [40,41]. An additional proof of high silencing efficiency was the change in ovarian phenotype. Ovarian development was blocked when the expression of ds*LsVg* or ds*LsVgR* was silenced. Either *LsVg* or *LsVgR* was required for ovarian development in female adults, and when ds*LsVg* or ds*LsVgR* were injected together, their combined effect was greater. A decreased expression of *Vg* and *VgR* resulted in a reduction in yolk accumulation in the ovaries. In *Liposcelis entomophila*, inadequately developed ovaries were observed after feeding ds*LeVgR* [42]. The injection of dsRNA, targeting the *VgR* of *Samia ricini* disrupted the reproductive morphology and lead to yolk deposition, a decrease in egg cell numbers, and ovarian malformation [43]. Reducing the expression of *Vg* in female adults of *Nilaparvata lugens* seriously inhibited the growth of oocytes in the ovary, and the oocytes were deformed and had irregular edges [31]. In addition, mating of normal *Chrysopa pallens* females with *Vg*-deficient males downregulated the expression of *Vg* in the females and inhibited the development of the ovaries [44]. These studies indicate that *Vg* and *VgR* play a critical role in the development of oocytes and are vital factors involved in female reproduction.

A high reproductive capacity is common in the majority of important pest species. This results in rapid population increase and enhanced crop damage. RNAi targeting *Vg* and *VgR* could be an effective way to restrict individual numbers of pest populations and reduce damage. In the present study, we found that silencing *LsVg* and *LsVgR* led to the malformation of the ovaries, a reduced oviposition period, fewer eggs, and lower hatching rates of *L. serricorne*. Similar effects have been observed in other insect species, such as *Cimex lectularius* [45], *Panonychus citri* [46], *Aphis citricidus* [36], *Cadra cautella* [47], *S. frugiperda* [11], and *Tuta absoluta* [12]. In addition, the co-silencing of *LsVg* and *LsVgR* led to a greater impact on the fecundity of *L. serricorne* and its reproductive period. The number of eggs produced and their hatching rate were significantly reduced compared with the single knockdown of *LsVg* or *LsVgR*. When multiple dsRNAs are simultaneously applied to the same insect to silence the expression of multiple target genes, the phenotype of RNAi can usually be enhanced [48]. In *N. lugens*, the co-silencing of *NlFer1* and *NlFer2* led to more obvious phenotypic changes during development [49]. When screening for genes lethal to *Chilo suppressalis*, the use of dsRNA mixtures targeting multiple genes significantly increased the mortality of larvae [50]. Based on these results, we suggest that a common silent target should be used in the application of RNAi to manage pests. However, this is only an initial step. Challenges exist for the efficient application of RNAi. In this study, we only tested the RNAi effect of *LsVg* and *LsVgR* by dsRNA injection. However, oral delivery is more practical when RNAi is used as a control method. In this case, we will need to establish an oral delivery system for *L. serricorne*. Another consideration is the poor stability and high cost of synthesized dsRNA, which seriously restricts the application of RNAi. Producing dsRNA using a prokaryotic expression system is an alternative way to reduce costs. Using nanoparticles as carriers of dsRNA would be helpful for increasing the stability of dsRNA in specific situations [51,52]. Additionally, it is important to evaluate the safety of dsRNA for non-target insects before it is widely used for pest control [53], and future research will test the effects of ds*LsVg* and ds*LsVgR* on natural enemies found in tobacco warehouses. *Vg* and *VgR* genes have always been efficient targets to control insect reproductive processes. The dsRNA targeting these genes can delay egg development, induce insect infertility, and cause the formation of inactive ovaries. These changes provide a potential way to manage pest populations.

## 5. Conclusions

In conclusion, we identified and characterized *LsVg* and *LsVgR* genes from the cigarette beetle *L. serricorne*. RNAi screening showed that the suppression of *LsVg* or *LsVgR* impaired ovarian development and decreased fecundity in *L. serricorne*. Co-silencing *LsVg* and *LsVgR* had a more significant effect on reducing the oviposition period, the hatching rate, and the number of eggs laid. These findings suggest that *LsVg* and *LsVgR* are integral to successful female reproduction and provide promising targets for developing an RNAi-based control method against *L. serricorne*.

## Data Availability

Data are contained within the article or Supplementary Materials.

## Supplementary Materials

The following supporting information can be downloaded at: https://www.mdpi.com/article/10.3390/insects16020175/s1, Table S1: Primers used in this study; Table S2: Information for insect species used in the phylogenetic analysis.
