# Supplementary material for: Roles of Vitellogenin and Its Receptor Genes in Female Reproduction of the Cigarette Beetle, Lasioderma serricorne
Source: Insects. 2025 Feb 6;16(2):175. doi: 10.3390/insects16020175 (PMC11857020; doi:10.3390/insects16020175)
Supplement: Supplementary file 1 [file insects-16-00175-s001.zip › insects-3442187-supplementary/Table S1-0108.pdf]

Table S1. Primers used in this study.

Gene name	Forward primer (5′-3′)	Reverse primer (5′-3′)	Application primers
LsVg	TAACCGGATTGAAGGACACC	TCGGCATCTTCTTACTAACC	ORF confirmation
LsVgR	TTTCAATCCGGACCTTTGTG	TCTTCTTAACTTCCCCCGGG	
LsVg	CGAACCGTCTCTTGCAGTTT	ATGGAAACCGTGAGCAACAG	qPCR analysis
LsVgR	CGAGCACTTTCCGTTGTTGA	CTCGCAATCTTTACTGGGCC	
Ls18s	GTTGATCACGTCGCAAGCTA	AGGTTTCCCTCTGGCTTGTT	
LsEF1α	GCATCTCCACGGATTTCACT	AAGGCAAGACGCTTATCGAA	
LsVg	**TAATACGACTCACTATAGGG**GTAAGTGTAGATGAATGGAAGGGA	**TAATACGACTCACTATAGGG**TACGGAATTCAATACTCCCAC	dsRNA synthesis
LsVgR	**TAATACGACTCACTATAGGG**CGGACGTGATGTAAAGTTTGTC	**TAATACGACTCACTATAGGG**AAACAACTTATACTGGTGCGACG	
GFP	**TAATACGACTCACTATAGGG**TACAAGACGCGTGCTGAAGT	**TAATACGACTCACTATAGGG**CAATGTTGTGGCGAATTTTG	

*The sequence in bold at the 5′end of the primer is the T7 promoter sequence.
