# Supplementary material for: Roles of Vitellogenin and Its Receptor Genes in Female Reproduction of the Cigarette Beetle, Lasioderma serricorne
Source: Insects. 2025 Feb 6;16(2):175. doi: 10.3390/insects16020175 (PMC11857020; doi:10.3390/insects16020175)
Supplement: Supplementary file 1 [file insects-16-00175-s001.zip › insects-3442187-supplementary/Table S2-0108-.pdf]

Table S2. Information for insect species used in the phylogenetic analysis.

Gene	Insect species	GenBank accession number	Family
Vitellogenin (Vg)	*Nilaparvata lugens*	BAF75351.1	Hemiptera
	Laodelphax striatellus	AGJ26478.1	
	Sogatella furcifera	QHD25542.1	
	Spodoptera litura	ABU68426.1	Lepidoptera
	Bombyx mori	NP_001037309.1	
	Helicoverpa armigera	XP_049705653.2	
	Papilio xuthus	KPJ04900.1	
	Nicrophorus vespilloides	XP_017781017.1	Coleoptera
	Anoplophora glabripennis	XP_018565741.1	
	Tribolium castaneum	XP_971398.2	
	Aethina tumida	XP_019873436.2	
	Harmonia axyridis	XP_045482401.1	
	Lasioderma serricorne	UVT84831.1	
	Aedes aegypti	AAA99486.1	Diptera
	Malaya genurostris	XP_058450293.1	
	Anopheles stephensi	XP_035895623.1	
	Blattella germanica	CAA06379.2	Blattaria
	Rhyparobia maderae	BAB19327.1	

	Periplaneta americana	XP_069693180.1	
Vitellogenin receptor (VgR)	*Bombyx mori*	NP_001184180.1	Lepidoptera
	Actias selene	AFV32171.1	
	Helicoverpa armigera	AGF33811.2	
	Spodoptera frugiperda	XP_035451670.2	
	Papilio xuthus	KPJ04221.1	
	Lasioderma serricorne	UVT84832.1	Coleoptera
	Aethina tumida	XP_019881581.2	
	Asbolus verrucosus	RZC40516.1	
	Colaphellus bowringi	AZN28756.1	
	Tribolium castaneum	XP_015837722.1	
	Tenebrio molitor	XP_068895053.1	
	Drosophila melanogaster	AAB60217.1	Diptera
	Aedes aegypti	AAC28497.1	
	Armigeres subalbatus	XP_062539894.1	
	Bactrocera dorsalis	ALA27368.1	
	Sogatella furcifera	QHI00364.1	Hemiptera
	Laodelphax striatellus	QBA97744.1	
	Nilaparvata lugens	XP_039295292.1	
	Blattella germanica	CAJ19121.1	

	Rhyparobia maderae	BAE93218.1	Blattaria
	Periplaneta americana	XP_069676025.1	
